# Volumetric brain MRI signatures of heart failure with preserved ejection fraction in the setting of dementia

**DOI:** 10.1016/j.mri.2024.02.016

**Published:** 2024-02-29

**Authors:** Camilo Bermudez, Cailey I. Kerley, Karthik Ramadass, Eric H. Farber-Eger, Ya-Chen Lin, Hakmook Kang, Warren D. Taylor, Quinn S. Wells, Bennett A. Landman

**Affiliations:** a Department of Biomedical Engineering, Vanderbilt University, Nashville, TN, USA; b Department of Electrical and Computer Engineering, Vanderbilt University, Nashville, TN, USA; c Department of Computer Science, Vanderbilt University, Nashville, TN, USA; d Department of Biostatistics, Vanderbilt University Medical Center, Nashville, TN, USA; e Department of Cardiology, Vanderbilt University School of Medicine, Nashville, TN, USA; f Department of Psychiatry, Vanderbilt University Medical Center, Nashville, TN, USA

**Keywords:** Heart failure, Dementia, Magnetic resonance imaging, Data mining, Electronic health record, Clinical imaging

## Abstract

Heart failure with preserved ejection fraction (HFpEF) is an important, emerging risk factor for dementia, but it is not clear whether HFpEF contributes to a specific pattern of neuroanatomical changes in dementia. A major challenge to studying this is the relative paucity of datasets of patients with dementia, with/without HFpEF, and relevant neuroimaging. We sought to demonstrate the feasibility of using modern data mining tools to create and analyze clinical imaging datasets and identify the neuroanatomical signature of HFpEF-associated dementia. We leveraged the bioinformatics tools at Vanderbilt University Medical Center to identify patients with a diagnosis of dementia with and without comorbid HFpEF using the electronic health record. We identified high resolution, clinically-acquired neuroimaging data on 30 dementia patients with HFpEF (age 76.9 ± 8.12 years, 61% female) as well as 301 age- and sex-matched patients with dementia but without HFpEF to serve as comparators (age 76.2 ± 8.52 years, 60% female). We used automated image processing pipelines to parcellate the brain into 132 structures and quantify their volume. We found six regions with significant atrophy associated with HFpEF: accumbens area, amygdala, posterior insula, anterior orbital gyrus, angular gyrus, and cerebellar white matter. There were no regions with atrophy inversely associated with HFpEF. Patients with dementia and HFpEF have a distinct neuroimaging signature compared to patients with dementia only. Five of the six regions identified in are in the temporo-parietal region of the brain. Future studies should investigate mechanisms of injury associated with cerebrovascular disease leading to subsequent brain atrophy.

## 1. Introduction

Dementia affects >5.3 million Americans and results in over $259 billion in medical expenditures annually [1]. Epidemiologic evidence suggests that heart failure with preserved ejection fraction (HFpEF) may be an independent risk factor for neurocognitive disease, including dementia disorders [2–4]. For example, patients with HFpEF have specific changes in neuropsychological testing, particularly in executive function, attention, and memory, as well as brain morphology compared to healthy adults [5–7]. Additionally, the proportion of vascular dementia to Alzheimer’s disease is increased among patients with HFpEF [8,9]. However, HFpEF and dementia also share many common risk factors such as advanced age, obesity, smoking, midlife hypertension, and diabetes [9,10]. It is unclear whether HFpEF confers dementia risk independently, in combination with, or through interactions with other comorbidities.

One approach to elucidating the relationship between HFpEF and dementia is to study the differences in brain structure using neuro-imaging, and an improved understanding of the imaging predictors of dementia in the setting of HFpEF may help characterize pathophysiologic mechanisms and lead to improved diagnostic and therapeutic strategies. Accessing the large volume of imaging and phenotypic data within electronic health records (EHR) could potentially allow for large-scale analyses in clinically relevant populations, but such studies have been limited by technical challenges related to systematically analyzing large volumes of non-standardized imaging studies. Recent advances in quantitative medical image processing and machine learning have made possible the discovery of imaging biomarkers, at scale, for disease prediction, detection, and surveillance [11,12]. For example, these efforts have demonstrated associations between decline in cerebral blood flow and higher Framingham Cardiovascular Disease Risk Profile (FCRP) score [13] as well as left ventricular mass index and changes in white matter microstructure [14].

We hypothesized that dementia in the setting of HFpEF would be associated with distinct comorbidity and brain morphology imaging signatures compared to dementia in the absence of HFpEF. To assess this HFpEF-dementia phenotype, we performed a case-control retrospective analysis of clinical diagnoses and brain imaging from 331 ambulatory patients with dementia from Vanderbilt University Medical Center (VUMC). We used a bioinformatics framework to identify and validate an HFpEF-dementia cohort and compare its clinical phenotype to that of dementia alone. We performed a volumetric analysis for changes in brain morphology associated with HFpEF-dementia identified on brain MRI. We also performed a PheWAS analysis to define the clinical comorbidities associated with HFpEF-dementia compared to dementia alone.

## 2. Method

### 2.1. Cohort Identification

**Fig. 1.**
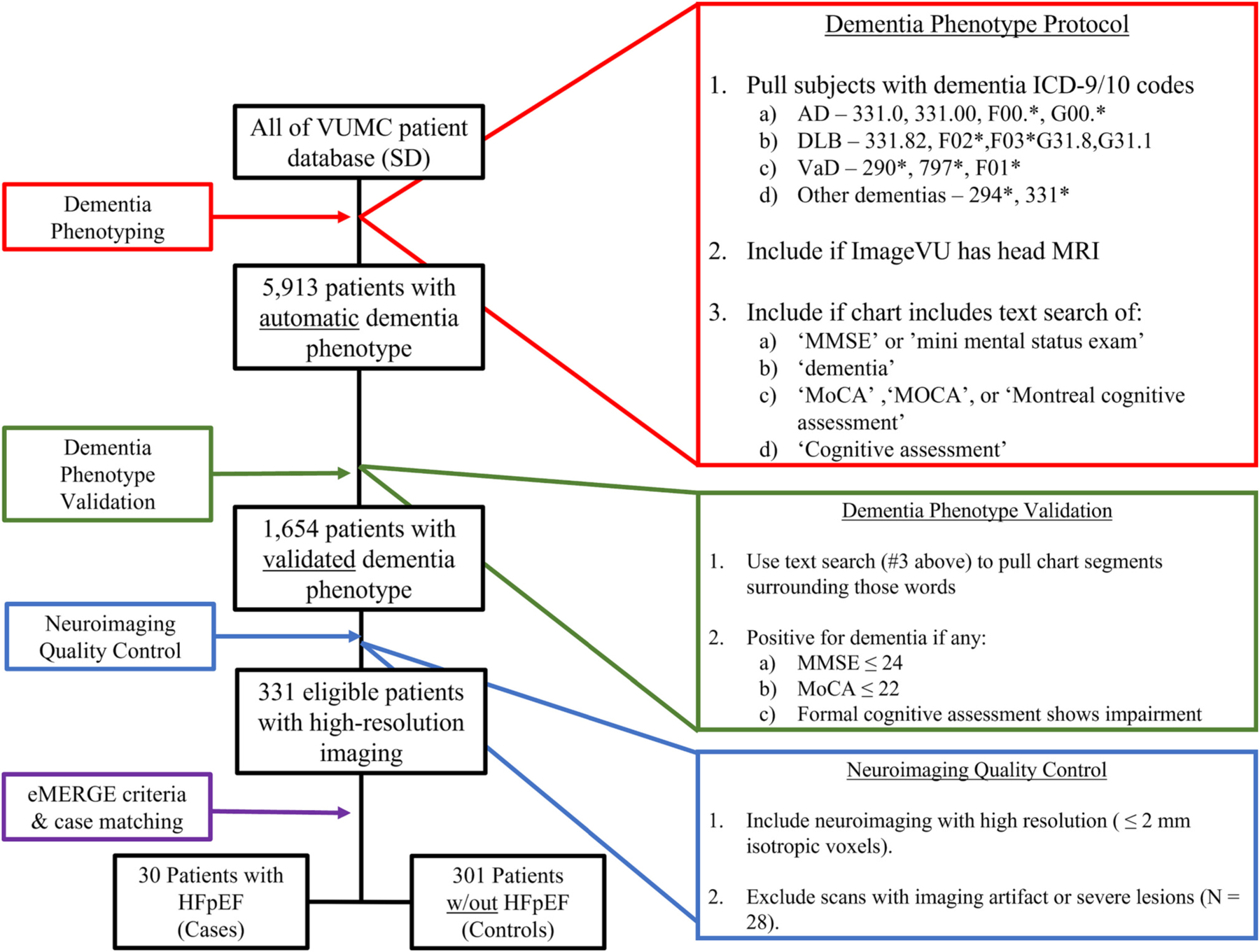
Inclusion and exclusion criteria protocol to identify patients with dementia and neuroimaging with and without heart failure.

The study cohort was derived from all subjects in the VUMC EHR. We first identified all possible subjects using a computable dementia algorithm that included both ICD-9 and ICD-10 codes (Fig. 1). We next limited the sample to patients with head MRI scans documented in the VUMC clinical imaging database ImageVU. Finally, we restricted the dataset to only patients with evidence of cognitive testing in their EHR. Specific strings used to identify cognitive testing in the chart are shown in the red box in Fig. 1.

The diagnosis of HFpEF was defined using the validated heart failure algorithm published by Bielinski et al. [15], which uses ICD-9 codes and problem list data, in combination with left ventricular ejection fraction data from clinical echocardiography reports. HFpEF was defined as clinically diagnosed HF with an echocardiographically documented LVEF ≥50%. This algorithm was manually validated on a subset of subject and demonstrated a positive predictive value of 95%. In order to validate the diagnosis of clinical dementia, we conducted text searches for cognitive testing to extract segments of the medical chart for manual review (Fig. 1). We used the following cutoffs to validate the diagnosis of dementia: a) mini-mental status exam score ≤ 24, b) Montreal Cognitive Assessment ≤22, or c) formal neuropsychological assessment showing cognitive impairment.

Last, we limited the sample to those patients with a high-quality brain MRI imaging. Quality assessment was performed by visual inspection to ensure that the studies retrieved were in fact brain MRIs without large artifacts that would limit structural analysis. The MRI scans were further sorted into T1-weighted brain MRI and T2-FLAIR brain MRI for subsequent studies. Only T1-weighted MRI with a resolution of 2.2 mm or finer were used.

### 2.2. Quantitative imaging analysis

The robust regression is critical as it helps achieve the expected linearity of score across slice indices. We performed an automatic segmentation protocol on all T1-weighted brain MRI using the spatially localized atlas network tiles (SLANT) segmentation algorithm [16]. The SLANT method integrates traditional medical image processing techniques with deep neural networks, distributing multiple independent 3D fully convolutional networks (FCN) for high-resolution whole brain segmentation. SLANT achieved high reproducibility across clinical MRI protocols [17], and it has successfully been adapted for pediatric and post-contrast MRI segmentation [18,19]. SLANT performs a whole brain segmentation into 132 cortical, subcortical, cerebellar, and white-matter regions of interest (ROI) following the BrainCOLOR labelling protocol. The volume of each ROI in mm^3^ was calculated by multiplying the number of labelled voxels by the voxel size. We used the automated library SIENAX from Freesurfer v6.0 to account for head size, which has been previously described and validated [20,21].

### 2.3. Statistical analysis

Two independent analyses were conducted: a PheWAS study to identify clinical variables associated with HFpEF-associated dementia and a volumetric MRI study to detect changes in regional brain volumes in HFpEF-associated dementia. The PheWAS method uses a validated, curated medical phenotypes (PheCodes) to rapidly identify phenome-wide association between exposures including clinical variation and disease phenotypes [22–24]. PheWAS codes are validated groupings of related ICD-9 (before October 2017) and ICD-10 (after October 2017) billing codes that capture the extended range of clinical diagnoses within an EHR data set.^23,24^ We used a logistic regression model to identify the relationships between PheCodes and the presence of HFpEF in the setting of dementia versus no HFpEF in patients with dementia while matching for sex and age at imaging. Of the total 1866 codes, we excluded phenotypes affecting a single gender or with ≤5% prevalence, which resulted in 397 clinical phenotypes included in the analyses. A Bonferroni correction was applied to account for multiple testing. The second analysis used logistic regression to assess relationships between volumetric estimates of ROI from T1-weighted MRI and exposure to heart failure in the setting of dementia. To standardize volumetric measurements across participants, we included the SIENAX scaling factor for intracranial volume as a covariate to adjust for head size. Associations with a false discovery rate q-value <0.05 were considered statistically significant. We evaluated the association between atrophy of brain regions and the presence of HFpEF using the log odds ratio. The odds of atrophy in a brain region compared to the odds of HFpEF was expressed as a ratio, the log of which was taken to linearize percentage changes.

## 3. Results

### 3.1. Characteristics of the identified cohorts

We identified 5913 subjects meeting criteria for an automated diagnosis of dementia, including 1092 (18.5%) with HFpEF (Fig. 1). The diagnosis of dementia was validated in 1654 patients, of which 393 (24%) had HFpEF. After selecting for patients with high-resolution imaging, the final cohort included 331 patients with dementia and high-resolution T1-weighted brain MRI, 30 (9%) of which met criteria for HFpEF.

Participant characteristics are provided in Table 1. All subjects with HFpEF and dementia were matched 10:1 to patients with dementia alone for age, sex, and image resolution. The mean age in the entire sample was 76.2 ± 8.48 years; 59% were women. The mean duration of dementia at time of imaging was 0.79 ± 1.75 years for cases and 1.15 ± 2.14 years for controls (*p* = 0.65; Wilcoxon rank-sum test) as indicated by the time between first incidence of dementia ICD code and imaging. The mean ejection fraction for patients with HFpEF was 54.7% +/– 10.2 as ascertained from the echocardiography report. After all inclusion criteria and matching, there were a total of 30 cases of HFpEF and dementia and 301 controls of dementia alone.

### 3.2. Anatomical volumetry differences associated with heart failure

**Fig. 2.**
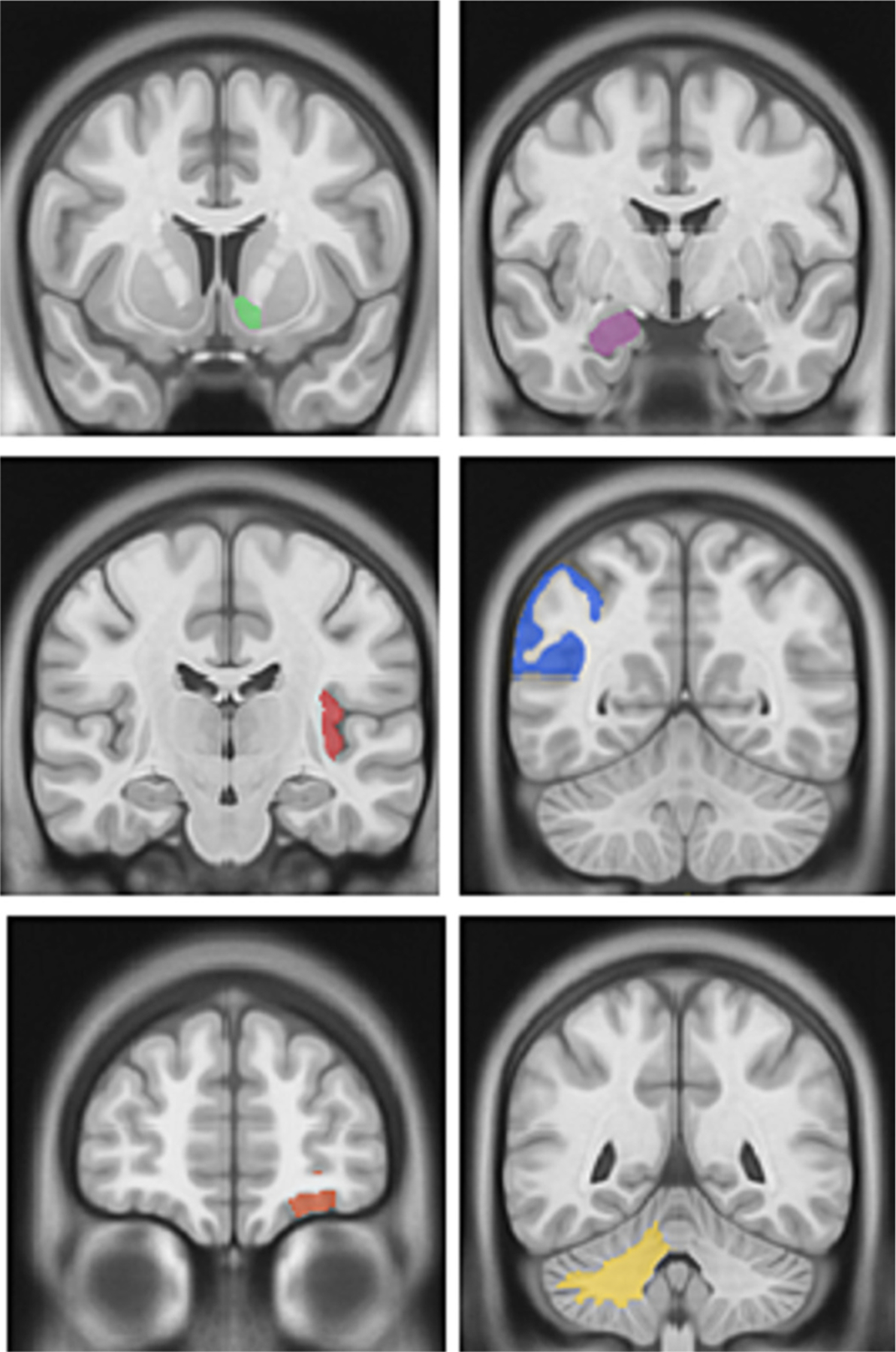
Significant brain regions with atrophy associated with HFpEF in the setting of dementia. Top left: Left Accumbens Area; Top right: Right Amygdala; Middle left: Left Posterior Insula; Middle right: Right Angular Gyrus; Bottom left: Left Anterior Orbital Gyrus; Bottom right: Right Cerebellar White Matter.

Brain volumes for six regions of interest were significantly different (q < 0.05) between dementia patients with and without HFpEF (Table 2). All of the significant regions showed atrophy associated with exposure to HFpEF. The six regions with volume loss associated with HFpEF were the left accumbens, right amygdala, left posterior insula, left anterior orbital gyrus, right angular gyrus, and right cerebellar white matter (Fig. 2). Five of these six significant regions are located in the temporo-parietal region of the brain.

**Fig. 3.**
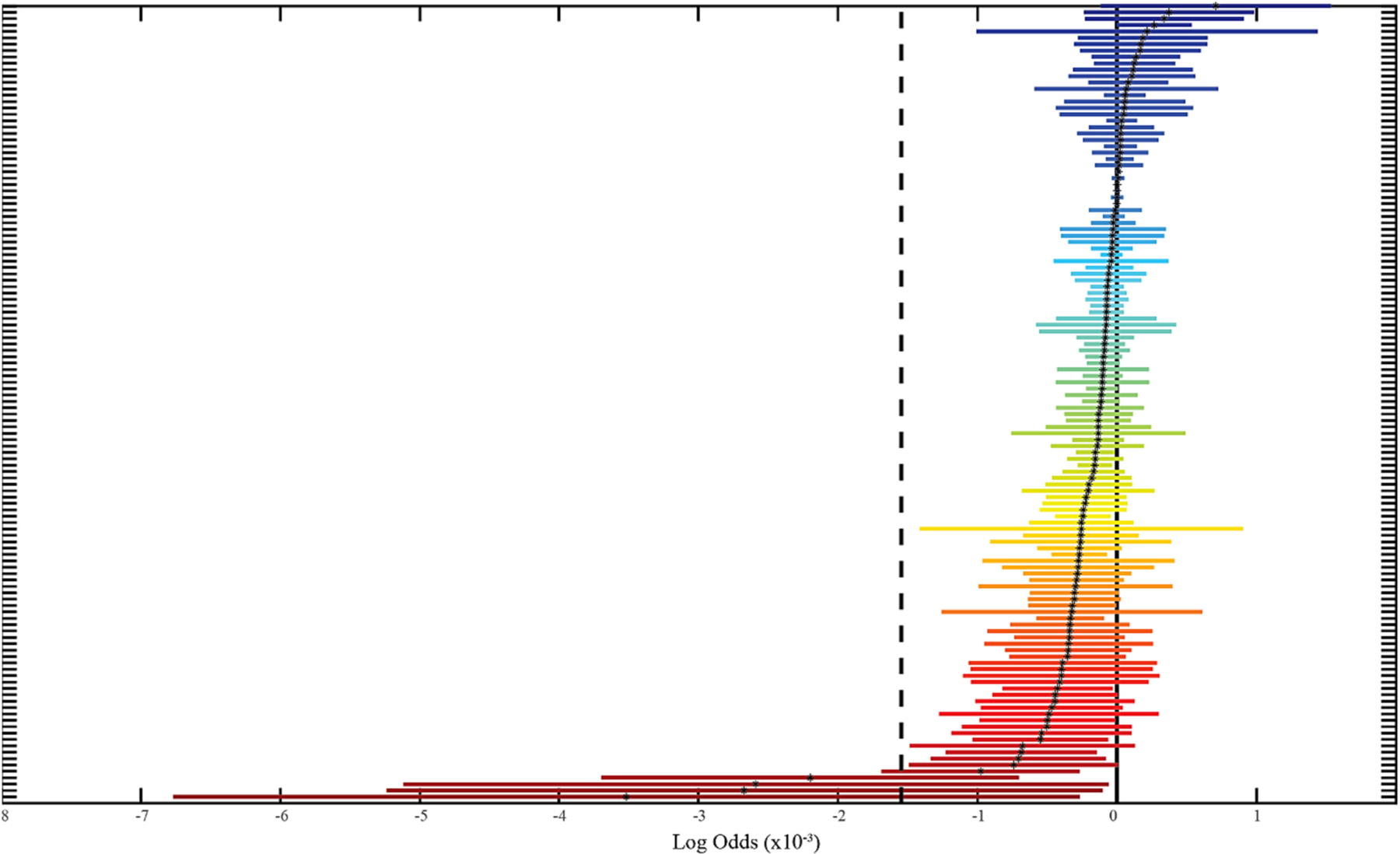
Volumetric analysis of non-significant regions. All non-significant regions are shown on the y-axis with the corresponding log odds and 95% confidence intervals. These regions are ranked according to predicted effect size, where negative effect size signifies volume loss associated with heart failure with preserved ejection fraction (HFpEF). A full table of all sorted regions and corresponding effect sizes is shown in Supplement Table 1.

Fig. 3 shows the results for all regions, sorted according to effect size. Note that only four regions have a larger effect size than the significant regions found in this study, and two of these correspond to the contralateral side of a significant structure. However, there are a large number of non-significant regions with atrophy where the lower bound of the confidence interval of the effect size is bounded by –1 × 10^-3^. Results for all regions are shown in the Supplementary Table 1.

### 3.3. Clinical comorbidities associated with heart failure

**Fig. 4.**
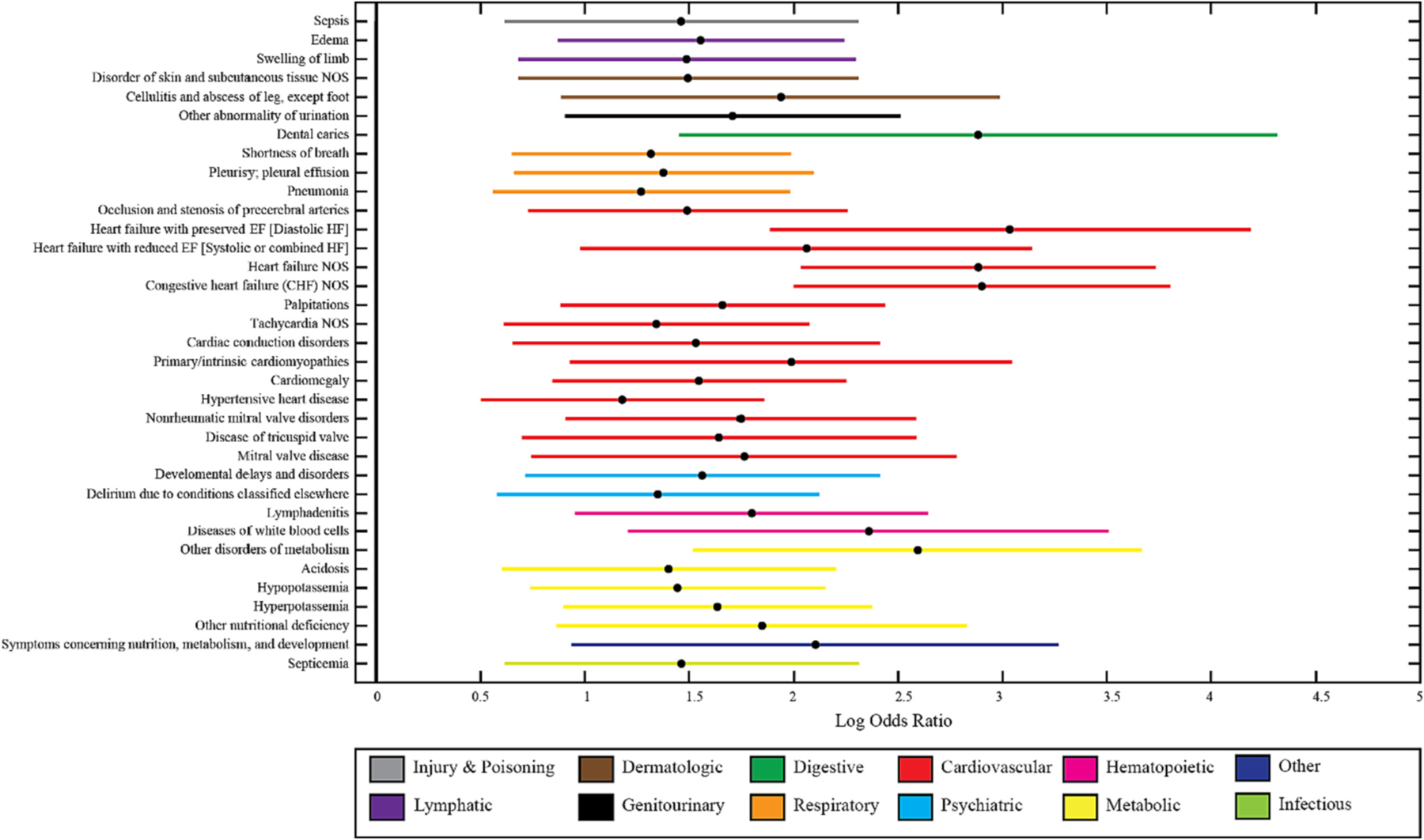
PheWAS analysis done on HFpEF dementia cohort. Significant clinical phenotypes associated with heart failure with preserved ejection fraction (HFpEF) in patients with dementia. Clinical phenomes are organized by organ system. A full table of significant clinical variables is shown in Supplement Table 2.

PheWAS analysis identified a large number of clinical comorbidities associated with HFpEF in the setting of dementia (Fig. 4). Not surprisingly, HFpEF with dementia was associated with a wide range of phenotypes dominated by cardiovascular disease and associated comorbidities, such as respiratory and metabolic abnormalities. All significant clinical phenotypes were positively associated (i.e., more common) with the presence of HFpEF.

## 4. Discussion and conclusion

We extracted clinical data with neuroimaging to identify the clinical comorbidities and changes in brain imaging morphology of HFpEF-associated dementia. Collectively, the findings suggest that HFpEF has a unique clinical and anatomic signature from dementia in the absence of HFpEF. Moreover, this study demonstrates the feasibility of mining large clinical datasets to identify unique clinical and radiological signatures.

Previous work has shown associations between cortical atrophy and cerebral hypoperfusion in the temporo-parietal watershed territories in Alzheimer’s disease [25,26]. Of these regions, atrophy of the accumbens area and the amygdala has been associated with HF previously, suggesting that HF may increase risk of Alzheimer’s disease through early insult to subcortical brain structures [27]. Similarly, atrophy of the insula [28] and the orbital gyrus [29] has been previously reported in vascular dementia while reduction of cerebral blood flow was shown in the angular gyrus in patients with Alzheimer’s disease [30]. Previous work has also shown atrophy of the cerebellar white matter is associated with heart failure when compared to controls [31]. Additionally, we observed a lateralizing effect on these structures. Previous work has shown a lateralizing effect on brain atrophy in semantic dementia, particularly on temporal lobe structures like the entorhinal cortex, amygdala, and fusiform gyrus [32] as well as the hippocampus in the context of HF [33]. These associations may require further study to understand the mechanism of lateral differences in brain volume and how they are related to HFpEF’s effect on brain perfusion and anatomy. A key limitation of these findings is that, given the total number of subjects available with high-resolution imaging from the EHR, the current study is underpowered to control for all possible confounders, such as the presence of hypertension, diabetes, and chronic kidney disease. While it is difficult to fully separate the effects of cardiovascular risk factors from HFpEF alone, we showed that HFpEF and its associated risk factors may have a unique signature on brain structural changes.

Importantly, the literature of brain structural changes associated with HF has focused on comparing patients with HF to healthy controls without taking into account the presence of clinical dementia [27,33,34]. In this work, we make similar observations to previous work, but focus on HF patients with dementia compared to controls with dementia. This suggests that the atrophy pattern identified may correspond to a HFpEF signature independent of the presence of dementia.

There are several potential mechanisms to explain our observation. HFpEF is frequently accompanied by diastolic dysfunction and an increase in left ventricular stiffness [35]. Left ventricular stiffness, in turn, coupled with arterial stiffness secondary to atherosclerotic disease and other comorbidities common in HFpEF, results in labile, pulsatile blood pressure, which has been associated with end-organ damage in organs with low microvascular resistance such as the brain and kidneys due to barotrauma and shear forces [36–39]. Moreover, aortic stiffness has been associated with all-cause dementia and Alzheimer’s disease [40], with a decrease in cerebral blood flow to the temporal lobe of subjects with cognitive deficits [41]. It is possible that the temporal brain regions identified in this study showing atrophy associated with HFpEF, such as the accumbens, amygdala, insula, and the angular gyrus, are preferentially susceptible to pulsatile flow and barotrauma [41]. This observation is also supported by the lack of significant atrophy in areas commonly associated with Alzheimer’s disease, such as the hippocampus, since the effect of dementia may be accounted for in both groups.

To explore the possibility of atrophy due to an HFpEF-dementia interaction effect, we considered the effect size of non-significant regions (Fig. 3). It is possible that a larger study would show significance in the contralateral regions with large effect size. Remarkably, many of the regions showed a trend towards atrophy associated with HFpEF, with the lower bound of the effect size being much smaller than the significant regions found in this study. This suggests that regions such as the hippocampus, parahippocampal gyrus, frontal cortex, and the entorhinal area are possible candidates for a specific HFpEF-dementia interaction atrophy, but this effect is much smaller than that of HFpEF. Future studies may seek to study these regions directly to identify interaction effects between HFpEF and dementia. Lastly, a limitation of this work is the absence of a HFpEF group without dementia. However, it is difficult to assume that clinical brain imaging acquired from patients with HFpEF would be cognitively normal. A prospective imaging study in patients with HFpEF and cognitive testing at the time of imaging would be necessary to account for brain changes due to HFpEF alone. Ultimately, we included only patients who had undergone a brain MRI in order to perform volumetric segmentations in this work. It is possible there are still a large number of patients with dementia and heart failure who have been diagnosed without acquiring neuroimaging. The clinical reasoning behind a lack of imaging, whether due to disease severity or other patient circumstances, may be a confounder in the description of a clinical phenotype as well as morphological brain changes.

Some of the most significant clinical comorbities found in our PheWAS study were associated with the cardiovascular system. This is not surprising, as HFpEF occurs in context of other cardiovascular disease and its risk factors, which may play a role in the development of dementia [5,42]. We also found previously reported HFpEF comorbidities in other organ systems, such as metabolic abnormalities, hematologic, and respiratory conditions [43]. A study to validate the presented systematic exploration in the context of PheWAS novelty [44] would be of interest. It is interesting that there were no clinical comorbidities inversely associated with HFpEF in the setting of dementia, suggesting that, in general, patients diagnosed with HFpEF are more medically ill than individuals with dementia without HFpEF, confirming a previously reported finding [45]. One limitation of our approach is the role of important cardiovascular comorbidities of dementia, such as the presence of hypertension, diabetes, or smoking status. Future work may take these variables into account in both clinical phenome regressions as well as volumetric regressions to further understand the specific role of HFpEF in dementia.

The protocol described here for phenotype selection shown in Fig. 1 uses a semi-automated method of data extraction and validation to identify a clinical phenotype. We presented the analysis of HFpEF in the setting of dementia as an example of the kinds of deep phenotyping that can be achieved with these tools. These methods can be used to identify other clinical phenotypes with scarce datasets and examine associations with related diseases. This presents an important step in translational medicine, where disease processes can be studied directly from patient populations instead of recruited studies. Additionally, it expands the scale at which a large population of patients can be queried to identify possible participants with an entire HER available for analysis, including both clinical and imaging variables. This would open possibilities for the recruitment of larger datasets of uncommon diseases or in populations with recruitment limitations, such as elderly or critically ill.

## Supplementary Material

supplemental data

## Figures and Tables

**Table 1:** Cohort demographics. Values represented as mean ± SD.

	HPpEF and Dementia (*N* = 30)	Dementia without Heart Failure (*N* = 301)	*p*-value
Age (years)	76.9 ± 8.12	76.2 ± 8.52	0.66^*^
Sex (% female)	60.7%	59.8%	0.93^*^
Dementia Duration at MRI (years)	0.82 ± 1.83	1.20 ± 2.23	0.52 ^*^
Heart Failure Duration at MRI (years)	3.29 ± 3.67	N/A	N/A
Hypertension (%)	100%	92.7%	0.75 ^+^
Diabetes (%)	53.6%	19.6%	0.03 ^+^

* Wilcoxon rank-sum test.

+ Fisher’s exact test.

**Table 2:** Brain regions with significant atrophy associated with the presence of HFpEF. q-values are *p*-values corrected for multiple comparisons using the Bejamini-Hochberg method for false discovery rate.

Region of Interest	Log Odds Ratio	q-value
Left Accumbens Area	−4.4 × 10^−3^	0.020
Right Amygdala	−2.5 × 10^−3^	0.020
Left Posterior Insula	−1.6 × 10^−3^	0.012
Left Anterior Orbital Gyrus	−1.4 × 10^−3^	<0.001
Right Angular Gyrus	−2.0 × 10^−4^	0.043
Right Cerebellar White Matter	−2.0 × 10^−4^	0.04
