# Supplementary material for: Volumetric brain MRI signatures of heart failure with preserved ejection fraction in the setting of dementia
Source: Magn Reson Imaging. Author manuscript; Available in PMC 2025 Apr 4. (PMC11969415; doi:10.1016/j.mri.2024.02.016)
Supplement: supplemental data [file NIHMS2062433-supplement-supplemental_data.docx]

# Supplementary Material

**Supplemental Table 1. Volumetric analysis of regions of interest in the brain. A negative log odds signifies volume atrophy associated with heart failure. P-values are corrected for multiple comparisons using the Benjamini-Hochberg false discovery rate.**

| **Region of Interest** | **Log Odds** | **Corrected**  **p-value** |
| --- | --- | --- |
| **Left Accumbens Area** | **-4.42E-03** | **0.024** |
| Left Basal Forebrain | -3.58E-03 | 0.204 |
| Right Basal Forebrain | -2.72E-03 | 0.220 |
| Right Accumbens Area | -2.62E-03 | 0.273 |
| **Right Amygdala** | **-2.46E-03** | **0.024** |
| Left Amygdala | -2.25E-03 | 0.065 |
| **Left posterior insula** | **-1.58E-03** | **0.012** |
| **Left anterior orbital gyrus** | **-1.37E-03** | **<0.001** |
| Left planum polare | -9.60E-04 | 0.108 |
| Right entorhinal area | -7.06E-04 | 0.204 |
| Right gyrus rectus | -6.86E-04 | 0.324 |
| Left parahippocampal gyrus | -6.80E-04 | 0.154 |
| Right Hippocampus | -6.41E-04 | 0.067 |
| Left medial frontal cortex | -6.31E-04 | 0.387 |
| Right occipital pole | -5.43E-04 | 0.204 |
| Left Hippocampus | -5.42E-04 | 0.204 |
| Right posterior insula | -5.35E-04 | 0.365 |
| Right parahippocampal gyrus | -5.32E-04 | 0.332 |
| Left gyrus rectus | -4.85E-04 | 0.472 |
| Left occipital pole | -4.84E-04 | 0.173 |
| Left Caudate | -4.71E-04 | 0.323 |
| Left lateral orbital gyrus | -4.63E-04 | 0.258 |
| Left postcentral gyrus medial segment | -4.60E-04 | 0.556 |
| Right orbital part of the inferior frontal gyrus | -4.54E-04 | 0.387 |
| Left entorhinal area | -4.42E-04 | 0.470 |
| Left subcallosal area | -4.22E-04 | 0.430 |
| Right medial frontal cortex | -4.08E-04 | 0.479 |
| Right frontal operculum | -4.06E-04 | 0.465 |
| Right occipital fusiform gyrus | -3.98E-04 | 0.181 |
| Right subcallosal area | -3.92E-04 | 0.516 |
| Left middle occipital gyrus | -3.55E-04 | 0.067 |
| Right calcarine cortex | -3.54E-04 | 0.204 |
| Left central operculum | -3.39E-04 | 0.332 |
| Left anterior insula | -3.27E-04 | 0.387 |
| Left orbital part of the inferior frontal gyrus | -3.25E-04 | 0.556 |
| Right anterior insula | -3.10E-04 | 0.387 |
| Cerebellar Vermal Lobules VIII-X | -3.05E-04 | 0.556 |
| Right temporal pole | -3.02E-04 | 0.065 |
| Right central operculum | -3.01E-04 | 0.422 |
| Right Caudate | -3.01E-04 | 0.422 |
| Left Thalamus Proper | -3.01E-04 | 0.387 |
| Left posterior orbital gyrus | -3.00E-04 | 0.448 |
| Right Thalamus Proper | -2.97E-04 | 0.332 |
| Left temporal pole | -2.93E-04 | 0.067 |
| Cerebellar Vermal Lobules I-V | -2.82E-04 | 0.332 |
| Right anterior orbital gyrus | -2.72E-04 | 0.556 |
| Left triangular part of the inferior frontal gyrus | -2.53E-04 | 0.360 |
| Right superior occipital gyrus | -2.38E-04 | 0.390 |
| Right transverse temporal gyrus | -2.22E-04 | 0.705 |
| **Right angular gyrus** | **-2.22E-04** | **0.043** |
| Right planum polare | -2.21E-04 | 0.705 |
| Left transverse temporal gyrus | -2.15E-04 | 0.705 |
| Left medial orbital gyrus | -2.10E-04 | 0.446 |
| Left cuneus | -2.08E-04 | 0.422 |
| Right frontal pole | -2.07E-04 | 0.422 |
| Left superior temporal gyrus | -2.05E-04 | 0.324 |
| Left frontal pole | -2.03E-04 | 0.448 |
| **Right Cerebellum White Matter** | **-1.94E-04** | **0.044** |
| Left lingual gyrus | -1.93E-04 | 0.322 |
| Right Ventral DC | -1.85E-04 | 0.679 |
| Right precentral gyrus | -1.66E-04 | 0.186 |
| Left Cerebellum White Matter | -1.59E-04 | 0.136 |
| Right lingual gyrus | -1.57E-04 | 0.360 |
| Right Pallidum | -1.56E-04 | 0.918 |
| Right superior temporal gyrus | -1.53E-04 | 0.448 |
| Left anterior cingulate gyrus | -1.53E-04 | 0.472 |
| Right anterior cingulate gyrus | -1.46E-04 | 0.526 |
| Left superior occipital gyrus | -1.43E-04 | 0.636 |
| Right triangular part of the inferior frontal gyrus | -1.36E-04 | 0.678 |
| Right planum temporale | -1.34E-04 | 0.777 |
| Right superior frontal gyrus medial segment | -1.24E-04 | 0.446 |
| Left angular gyrus | -1.24E-04 | 0.323 |
| Left calcarine cortex | -1.14E-04 | 0.705 |
| Brain Stem | -1.13E-04 | 0.322 |
| Left superior frontal gyrus medial segment | -1.10E-04 | 0.551 |
| Left postcentral gyrus | -1.10E-04 | 0.390 |
| Right posterior cingulate gyrus | -1.09E-04 | 0.705 |
| Left middle temporal gyrus | -1.08E-04 | 0.322 |
| Left supramarginal gyrus | -1.04E-04 | 0.422 |
| Right inferior temporal gyrus | -1.01E-04 | 0.391 |
| Right superior parietal lobule | -9.40E-05 | 0.397 |
| Left Ventral DC | -9.33E-05 | 0.841 |
| 4th Ventricle | -9.16E-05 | 0.779 |
| Left precentral gyrus | -8.74E-05 | 0.472 |
| Right cuneus | -8.71E-05 | 0.705 |
| Left opercular part of the inferior frontal gyrus | -8.04E-05 | 0.779 |
| Right precuneus | -7.81E-05 | 0.556 |
| Left supplementary motor cortex | -7.65E-05 | 0.705 |
| Left inferior temporal gyrus | -7.63E-05 | 0.465 |
| Right middle temporal gyrus | -7.15E-05 | 0.479 |
| Right inferior occipital gyrus | -6.68E-05 | 0.679 |
| Right middle frontal gyrus | -4.77E-05 | 0.477 |
| Right medial orbital gyrus | -4.55E-05 | 0.920 |
| Left middle frontal gyrus | -3.95E-05 | 0.556 |
| Right supramarginal gyrus | -3.93E-05 | 0.779 |
| Right postcentral gyrus | -3.83E-05 | 0.779 |
| Left posterior cingulate gyrus | -3.82E-05 | 0.943 |
| Left occipital fusiform gyrus | -2.73E-05 | 0.946 |
| Right parietal operculum | -2.32E-05 | 0.964 |
| Left inferior occipital gyrus | -3.78E-06 | 0.982 |
| Right Cerebral White Matter | -3.29E-06 | 0.705 |
| Right fusiform gyrus | -1.70E-06 | 0.994 |
| Left Cerebral White Matter | -7.72E-07 | 0.950 |
| Right precentral gyrus medial segment | -4.53E-07 | 0.999 |
| Left Lateral Ventricle | -3.97E-07 | 0.982 |
| Right Cerebellum Exterior | 3.90E-06 | 0.946 |
| Left superior parietal lobule | 8.49E-06 | 0.957 |
| Right Lateral Ventricle | 9.03E-06 | 0.556 |
| Left Cerebellum Exterior | 1.10E-05 | 0.781 |
| Left Inf Lat Vent | 1.28E-05 | 0.978 |
| Right middle occipital gyrus | 1.29E-05 | 0.964 |
| Right supplementary motor cortex | 1.38E-05 | 0.964 |
| Right superior frontal gyrus | 1.62E-05 | 0.884 |
| Left fusiform gyrus | 2.13E-05 | 0.943 |
| Left planum temporale | 2.21E-05 | 0.964 |
| Left superior frontal gyrus | 3.16E-05 | 0.748 |
| Right lateral orbital gyrus | 4.30E-05 | 0.943 |
| Left precuneus | 5.51E-05 | 0.701 |
| Left parietal operculum | 8.12E-05 | 0.852 |
| 3rd Ventricle | 8.49E-05 | 0.740 |
| Cerebellar Vermal Lobules VI-VII | 9.06E-05 | 0.918 |
| Right opercular part of the inferior frontal gyrus | 1.03E-04 | 0.705 |
| Right middle cingulate gyrus | 1.04E-04 | 0.705 |
| Right posterior orbital gyrus | 1.50E-04 | 0.705 |
| Left Putamen | 2.01E-04 | 0.647 |
| Right Putamen | 2.15E-04 | 0.604 |
| Right Inf Lat Vent | 2.24E-04 | 0.556 |
| Left middle cingulate gyrus | 2.42E-04 | 0.332 |
| Left Pallidum | 2.49E-04 | 0.838 |
| Left frontal operculum | 2.69E-04 | 0.628 |
| Left precentral gyrus medial segment | 3.31E-04 | 0.505 |
| Right postcentral gyrus medial segment | 8.24E-04 | 0.291 |

**Supplemental Table 2. PheWAS results for significant clinical associations with HFpEF in the setting of dementia in patients with high resolution imaging. P-values are corrected for multiple comparisons using Bonferroni corrections.**

| **PheCode** | **Effect Size** | **95% Confidence Interval** | | **Corrected p-value** |
| --- | --- | --- | --- | --- |
| Septicemia | 1.87 | 0.95 | 2.79 | 0.028 |
| Type 1 diabetes | 2.10 | 1.11 | 3.09 | 0.013 |
| Type 2 diabetes with neurological manifestations | 2.47 | 1.47 | 3.48 | 0.001 |
| Polyneuropathy in diabetes | 2.49 | 1.37 | 3.61 | 0.005 |
| Disorders of lipoid metabolism | 1.85 | 0.99 | 2.72 | 0.010 |
| Hyperpotassemia | 1.97 | 1.11 | 2.83 | 0.003 |
| Hypopotassemia | 1.76 | 0.96 | 2.57 | 0.007 |
| Iron deficiency anemias, unspecified or not due to blood loss | 1.89 | 1.02 | 2.77 | 0.009 |
| Other anemias | 1.71 | 0.89 | 2.53 | 0.017 |
| Anemia of chronic disease | 2.34 | 1.17 | 3.51 | 0.035 |
| Alteration of consciousness | 1.75 | 0.94 | 2.55 | 0.008 |
| Mitral valve disease | 3.27 | 2.03 | 4.52 | <0.001 |
| Nonrheumatic mitral valve disorders | 2.79 | 1.81 | 3.77 | <0.001 |
| Nonrheumatic aortic valve disorders | 2.24 | 1.27 | 3.22 | 0.003 |
| Hypertensive heart and/or renal disease | 2.54 | 1.31 | 3.76 | 0.019 |
| Hypertensive heart disease | 2.54 | 1.63 | 3.45 | <0.001 |
| Unstable angina (intermediate coronary syndrome) | 2.35 | 1.37 | 3.34 | 0.001 |
| Myocardial infarction | 2.41 | 1.53 | 3.29 | <0.001 |
| Angina pectoris | 2.57 | 1.49 | 3.66 | 0.001 |
| Coronary atherosclerosis | 2.07 | 1.21 | 2.93 | 0.001 |
| Other chronic ischemic heart disease, unspecified | 3.14 | 2.21 | 4.07 | <0.001 |
| Other forms of chronic heart disease | 1.89 | 0.95 | 2.82 | 0.029 |
| Cardiomegaly | 2.44 | 1.58 | 3.29 | <0.001 |
| Nonspecific chest pain | 2.30 | 1.28 | 3.33 | 0.004 |
| Precordial pain | 1.79 | 0.89 | 2.70 | 0.042 |
| Cardiac conduction disorders | 2.03 | 1.00 | 3.05 | 0.044 |
| Atrial fibrillation | 2.32 | 1.44 | 3.21 | <0.001 |
| Tachycardia NOS | 1.65 | 0.82 | 2.49 | 0.041 |
| Atherosclerosis of aorta | 1.79 | 0.88 | 2.70 | 0.047 |
| Hypotension NOS | 1.99 | 1.15 | 2.84 | 0.002 |
| Acute bronchitis and bronchiolitis | 1.73 | 0.88 | 2.59 | 0.027 |
| Obstructive chronic bronchitis | 2.34 | 1.19 | 3.50 | 0.028 |
| Pleurisy; pleural effusion | 1.93 | 1.09 | 2.78 | 0.003 |
| Respiratory failure | 2.33 | 1.30 | 3.37 | 0.004 |
| Shortness of breath | 2.08 | 1.22 | 2.95 | 0.001 |
| Other dyspnea | 1.64 | 0.84 | 2.44 | 0.025 |
| Diarrhea | 1.84 | 1.02 | 2.66 | 0.004 |
| Acute renal failure | 1.62 | 0.81 | 2.43 | 0.034 |
| Disorder of skin and subcutaneous tissue NOS | 1.73 | 0.86 | 2.61 | 0.042 |
| Swelling of limb | 2.41 | 1.53 | 3.29 | <0.001 |
| Edema | 2.05 | 1.22 | 2.88 | 0.001 |
| Sepsis | 2.09 | 1.17 | 3.00 | 0.003 |
